# Terror Attacks Increase the Risk of Vascular Injuries

**DOI:** 10.3389/fpubh.2014.00047

**Published:** 2014-05-30

**Authors:** Eitan Heldenberg, Adi Givon, Daniel Simon, Arie Bass, Gidon Almogy, Kobi Peleg

**Affiliations:** ^1^Department of Vascular Surgery, Assaf Harofeh Medical Center, Sackler Faculty of Medicine, Tel Aviv University, Tel Aviv, Israel; ^2^National Center for Trauma and Emergency Medicine Research, The Gertner Institute for Health Policy and Epidemiology, Tel Hashomer, Israel; ^3^Trauma Unit, Sheba Medical Center, Sackler Faculty of Medicine, Tel Aviv University, Tel Aviv, Israel; ^4^Trauma Unit, Hadassah Medical Center, Hebrew University, Jerusalem, Israel; ^5^Department of Disaster Medicine, School of Public Health, Tel Aviv University, Tel Aviv, Israel

**Keywords:** terror-related trauma, suicide bombers, gun shot wounds, blast, improvised explosive device

## Abstract

**Objectives:** Extensive literature exists about military trauma as opposed to the very limited literature regarding terror-related civilian trauma. However, terror-related vascular trauma (VT), as a unique type of injury, is yet to be addressed.

**Methods:** A retrospective analysis of the Israeli National Trauma Registry was performed. All patients in the registry from 09/2000 to 12/2005 were included. The subgroup of patients with documented VT (*N* = 1,545) was analyzed and further subdivided into those suffering from terror-related vascular trauma (TVT) and non-terror-related vascular trauma (NTVT). Both groups were analyzed according to mechanism of trauma, type and severity of injury and treatment.

**Results:** Out of 2,446 terror-related trauma admissions, 243 sustained TVT (9.9%) compared to 1302 VT patients from non-terror trauma (1.1%). TVT injuries tend to be more complex and most patients were operated on. Intensive care unit admissions and hospital length of stay was higher in the TVT group. Penetrating trauma was the prominent cause of injury among the TVT group. TVT group had a higher proportion of patients with severe injuries (ISS ≥ 16) and mortality. Thorax injuries were more frequent in the TVT group. Extremity injuries were the most prevalent vascular injuries in both groups; however NTVT group had more upper extremity injuries, while the TVT group had significantly much lower extremity injuries.

**Conclusion:** Vascular injuries are remarkably more common among terror attack victims than among non-terror trauma victims and the injuries of terror casualties tend to be more complex. The presence of a vascular surgeon will ensure a comprehensive clinical care.

## Introduction

Bombs and explosions directed against civilian population are the primary instrument of global terrorism, resulting in death, injury, fear, and chaos. With the lessening of full scale military conflicts, terrorism has become a prominent feature of modern life, as manifested by the 300% raise in the number of serious incidents in recent years. Conventional explosives, available and easily accessible, are the most prevalent tools of terrorist attacks (1).

Although there is a very extensive literature relating to military trauma (2), the literature regarding terror-related trauma is insufficient, most of it originating from Israel due to the experience gained from suicide bombings throughout the country in recent years (1, 3–10). In Israel, two main methods were used by the terrorists-explosions, mainly by suicide bombers (SB), and gunshot wounds (GSW).

Suicide bombers explosions were targeted toward three main locations – buses, semi-confined spaces (covered open markets, restaurants or indoor cafés, night clubs, hotels), and open spaces (outdoor cafés, bus stops, and open markets) (10).

Explosions victims may present with varied patterns of injuries. In addition to the classic manifestations of blunt, penetrating injuries, and burns they may present with the unique pattern of blast injuries. Projectiles such as steel balls, nails, screws, and nuts packed around the explosive substance, a method frequently used by terrorists in Israel, caused secondary penetrating injuries. Multiple penetrations of such pellets resulted in devastating injuries and increased mortality (1, 3). Vascular trauma (VT) constitutes a very substantial part of the above mentioned injuries, most often threatening the patient’s life, extremity viability, or both.

Military trauma is characterized by high velocity missiles causing extensive soft tissue destruction, often involving multiple organ systems. These, as well as incidents affecting large number of casualties are no longer unique to military trauma and are present in terror trauma (TT) as well (3–5). Terrorist attacks generate both high velocity (>850 m/s) gun shot wounds, and shrapnel injuries from SB, which have the attributes of both high velocity and low velocity pellets, causing direct and indirect arterial injuries (12–15).

During the years 1987–1992 and once again between September 2000 and December 2005, the Israeli civilian population was exposed to multiple intensive terrorist attacks. The second wave of terrorist attacks was characterized by an extensive use of explosives, mainly improvise explosive devices (IED). These were usually ordinary explosive material strengthened by a large amount of nails and other metal shrapnel, producing an enhanced devastating killing power, in relation to the basic weight of the explosive material. Our aim was to analyze the data regarding VT due to terror attacks and to learn if there is any difference in the amount and pattern of injuries as compared to the usual civilian VT.

## Materials and Methods

A retrospective analysis of the Israeli Trauma Registry, gathered by the National Center for Trauma and Emergency Medicine Research in the Gertner Institute, was performed. At the time of the analysis, the Israeli trauma registry was based on information gathered from all six level-1 trauma centers in Israel and large four level 2-trauma centers, spread all over the country.

Every patient record in the registry is composed of around 200 different parameters such as demographic data, injury circumstances, transfer to trauma bay, diagnoses (ICD9, AIS), injury severity score (ISS), procedures performed, admission to intensive care unit (ICU), hospitalization length of stay (LOS), and outcome.

All patients registered from September 2000 to December 2005 were included in this analysis. We present here the subgroup of patients with documented VT (*N* = 1545). This subgroup was further subdivided into those patients suffering from terror-related vascular trauma (TVT) and non-terror-related vascular trauma (NTVT). Both groups were analyzed according to the type of trauma, as well as to type and severity of injury and treatment.

Statistical analysis was performed using SAS (SAS, Cary, NC, USA, version 9.1.3), and based mainly on descriptive statistics and group comparisons. Statistical tests included Pearson’s chi-square test for categorical data and the Wilcoxon non-parametric tests for continuous variables, which were not normally distributed. A *p* value <0.05 was considered statistically significant.

## Results

### General

During the period of September 2000 to December 2005, 122,208 trauma admissions were reported at the National Trauma Registry of Israel. Among those admissions, only 1,545 patients (1.3%) had vascular injuries, suffering from 1,626 vascular injuries (several patients’ sustained more than one vascular injury). There were 2,466 victims of terrorist attacks, of which 243 (9.85%) patients suffered VT (TVT), a highly significant difference in prevalence compared to 1302 VT patients from non-terror trauma (NTVT) (1.1%) (*p* < 0.01).

General data regarding the study population are presented in Table 1. Most of the VT patients in both subgroups were males. The majority of the VT patients in the TVT group were in the age group of 15–29 (58.8%), compared to 42.3% in the NTVT group (Table 1).

**Table 1:** **Vascular trauma – demographic and injury data – TVT vs. NTVT groups (%, number)**.

	Terror trauma (TVT) *N* = 243	Non-terror trauma (NTVT) *N* = 1302	*p* Value
**GENDER**			
Male	78.2% (190)	81.8% (1065)	NS
Female	21.8% (53)	18.1% (236)	
Unknown	0.0% (0)	0.1% (1)	
**AGE**			
0–14	4.5% (11)	14.1% (183)	<0.0001
15–29	58.8% (143)	42.3% (551)	<0.0001
30–44	23.1% (56)	23.7% (308)	NS
45–59	8.6% (21)	10.9% (142)	NS
60–74	2.5% (6)	5.4% (71)	0.0497
75+	0.8% (2)	2.9% (38)	NS
Unknown	1.7% (4)	0.7% (9)	
**ADDITIONAL DIAGNOSES**			
VT + other	96.3% (234)	88.5% (1152)	0.0002
VT only	3.7% (9)	11.5% (150)	
**ISS**			
1–8	7.0% (17)	29.3% (382)	<0.0001
9–14	29.6% (72)	29.1% (379)	NS
16–24	19.0% (46)	14.8% (192)	NS
25+	44.4% (108)	26.7% (347)	<0.0001
Unknown	0.0% (0)	0.1 (2)	

Almost all the TVT patients (96.3%) had other injuries, compared to 88.5% in the NTVT group. Injuries of TVT patients tend to be more complex and most of these patients were transferred to the trauma bay (72.8%), compared to 50.8% in the NTVT group (Table 2). ICU admissions were also significantly higher in the TVT group (58.0%) compared to the NTVT group (30.9%) (*p* < 0.0001).

**Table 2:** **Vascular trauma – treatment and outcome data – TVT vs. NTVT groups (%, number)**.

	Terror trauma (TVT) *N* = 243	Non-terror trauma (NTVT) *N* = 1302	*p* Value
Trauma bay on admission	72.8% (177)	50.8% (661)	<0.0001
ICU^a^ admission	58.0% (141)	30.9% (402)	<0.0001
Operation	89.7% (218)	79.1% (1030)	0.0001
LOS^b^ (mean of days + S.D)	18.3 + 18.1	11.7 + 16.3	<0.0001
Mortality	22.2% (54)	12.2% (159)	<0.0001

^a^ ICU, intensive care unit;

*^b^ LOS, length of stay*.

Vascular trauma was found to be a predictor of the need for surgical intervention. 89.7% of the TVT patients compared to 79.1% of the NTVT patients were operated (*p* < 0.0001) (Table 1).

Hospital LOS was significantly longer among the TVT patients compared to NTVT patients (18.3 ± 18.1 vs. 11.7 ± 16.3 days, respectively) (*p* < 0.0001) (Table 2).

While blunt trauma accounts for most of the injuries in the whole group of trauma patients, penetrating injury is the leading cause amongst VT group. This is even more prominent in the TVT group, where penetrating and combined penetrating and blunt trauma account for 93% of the cases, compared to 51.5% in the NTVT group (Table 3). Injuries sustained during terrorist attacks were either due to GSW (116 patients, 48%), explosions (109 patients, 45%), or stabbings and other causes (18 patients, 7%). The proportion of patients with severe injuries (ISS > 16) was higher in the TVT group compared to NTVT group (63.4 vs. 41.5%, respectively, *p* < 0.0001) (Table 1). Mortality followed the same pattern, 22.2% among the TVT patients vs. 12.2% among NTVT patients (*p* < 0.0001) (Table 2).

**Table 3:** **Vascular trauma – mechanism of injury – TVT vs. NTVT groups (%, number)**.

Trauma mechanism	TVT, *N* = 243	NTVT, *N* = 1302	*p* Value
Blunt	6.6% (16)	48.5% (631)	<0.0001
Penetrating	79.4% (193)	47.7% (621)	<0.0001
Combined blunt and penetrating	14.0% (34)	3.8% (50)	<0.0001

### Body regions of blood vessels injuries

The proportion of head and neck injuries was similar in the TVT group and in the NTVT group (18.9 and 16.4%, respectively, NS) (Table 4). Abdomen and pelvis injuries were also found in similar proportions in both groups (16.1 and 19.9%, respectively, NS). Thorax injuries were more frequent in the TVT group (16.5 vs. 11.8%, respectively, *p* = 0.05).

**Table 4:** **Body regions of vascular trauma and prominent injured vessels – TVT vs. NTVT groups (%, number)**.

Body region^a^	Terror trauma (TVT) *N* = 243	Non-terror trauma NTVT (*N* = 1302)	*p* Value
Head and neck	18.9 (46)	16.4 (214)	NS
Carotid artery	4.9 (12)	3.5 (45)	NS
Thorax	16.5 (40)	11.8 (154)	0.05
Thoracic aorta	5.4 (13)	6.1 (79)	NS
Innominate/subclavian arteries	4.9 (12)	0.8 (10)	<0.0001
Abdomen and pelvis	16.1 (39)	19.9 (259)	NS
Inferior vena cava	4.9 (12)	3.5 (46)	NS
Iliac vessels	4.1 (10)	5.0 (65)	NS
Upper extremity	18.1 (44)	35.5 (462)	<0.0001
Brachial vessels	7.8 (19)	5.8 (76)	NS
Radial vessels	2.1 (5)	11.0 (143)	<0.0001
Ulnar vessels	4.9 (12)	13.4 (175)	0.0002
Lower extremity	38.7 (94)	18.1 (236)	<0.0001
Femoral artery	16.5 (40)	5.3 (69)	<0.0001
Femoral veins	9.5 (23)	2.9 (38)	<0.0001
Popliteal vessels unspecified	2.1 (5)	1.5 (20)	NS
Popliteal artery	7.4 (18)	3.7 (48)	0.009
Popliteal vein	4.9 (12)	0.8 (10)	<0.0001
Multiple regions vascular injuries	8.2 (20)	1.7 (23)	<0.0001

*^a^ Patients might be injured in more than one region*.

Extremity injuries were the most prevalent vascular injuries, both among the TVT and the NTVT groups, 55.1 and 53.4%, respectively (NS). However, upper extremity injuries were much more common among the NTVT group, 35.5 vs. 18.1% in the TVT group (*p* < 0.0001), while lower extremity injuries were the most common injuries among the TVT group, 38.7% vs. only 18.1% in the NTVT group (*p* < 0.0001) (Table 4).

Multiple regions involvement was more prevalent among the TVT group than the NTVT group (8.2 vs. 1.7%, respectively, *p*<0.0001) (Table 4).

## Discussion

Terrorism, once considered as an isolated problem of developing countries, has evolved into a worldwide threat (1, 2). The September 11, 2001 coordinated attacks on the US, the bombing of the Madrid trains, the suicide bombing attacks on the public transportation in London, the bombings in Iraq, Afghanistan, Pakistan, Chechnya, the second Intifada in Israel, and more has exposed the world to the devastating effects of suicide bombing attacks (6, 10–12).

The number and extent of worldwide suicide attacks has risen sharply in recent years (9, 10, 16). Terrorist attacks against civilians have set up a new challenge to the medical and trauma teams. In contrary to non-vascular civilian trauma, which is mainly secondary to blunt mechanism, most of the civilian VT is secondary to penetrating injuries.

Terror-related injuries, which were caused by suicide bombers, wearing a suicide vest loaded with explosive material, and packed with multiple screws, nails, and other sharp objects, which magnifies the killing power, confronted the trauma teams and the vascular surgeons with a new type of injuries.

The suicide attackers mingle within the crowd and detonate those IED in the vicinity of their victims. The injuries sustained by the survivors of these attacks of “human bombs” combine the lethal effects of blast, penetrating and blunt mechanisms as well as burns (3, 9, 10, 16–21). Those bomb explosions resulted in about 20% on-scene mortality, while the survivors sustained blast (25%), penetrating shrapnel injuries (20–45%) or burns (15%) (2, 3, 5, 7, 10, 12).

According to our data, there was a much higher prevalence of vascular injuries in the terror settings (TVT) 10%, as compared to the non-terror scenarios (NTVT) 1%. Why is it so? This might be related to the fact that most of the NTVT injuries were caused by a single source, which course can usually be predicted. On the other hand in the TVT settings, injuries (which were either secondary to bombs or GSW) were caused, most of the times, by multiple sources. The bombs’ destructive power is much greater than the usual low velocity weapons such as handguns, knives, and even shotguns being used in most civilian settings, since it combines blast wave, originating from the explosion (primary blast injury), with penetrating, secondary and tertiary blast injuries (22). This might explain the higher percentage of VT among the TVT victims compared with NTVT victims.

Projectiles, like nails and metal balls embedded in the IED, following the rules of ballistics, will act as high or low velocity missiles and their injury pattern will reflect their size and shape. Different types of injury patterns have been defined for spherical missiles, nails, and screws. Since the shrapnel’s behavior cannot be predicted, as mentioned above, the injury pattern is not predictable. Multiple projectiles, causing penetrating injuries, combined with the tertiary blast injury, which throws the victim against solid objects, tend to cause larger tissue damage and injuries, with a higher degree of ISS and are more complex to treat (23).

The massive tissue damage resulting in either hemodynamic instability or severe ischemia, necessitate emergency vascular intervention. This may be an explanation for the low success rate of conservative treatment. Similar reports, regarding the severity of injury in the setting of terrorist bombing, were previously described (1, 23). The number of body regions injured was significantly increased in terror victims compared to the non-terror setting (1). In hospital, death among victims of explosions was only 4% while among GSW it was 22.8%. This probably reflects the instant death of the explosions victims, mainly those who were in close proximity to the center of the explosion, which, in confined places, can reach 29% (23).

The type of bomb (demolition charge left on the floor or suicide vest on the SB torso) was not specified in the registry, but it makes sense that those who survived were mainly hit by steel balls, which lose a lot of kinetic energy in their angle of flight, due to their round shape and increased air resistance. Their reduced velocity at the time of impact leads to large superficial tissue damage but limited penetration to deeper tissues. Nails, on the other hand, fly with better efficiency and cause much more internal damage.

Although the exact location of the injured patients during the explosion is unknown, it stands to reason that those who survived were not in close proximity to the center of the explosion.

The high frequency of upper extremity injuries, in the NTVT setting can be explained by their frequent use as protection to the face and neck. This pattern of injury changes dramatically in the TVT setting, toward injuries to the lower extremity. We assume that most of the injured patients who were hit in the torso region died on scene, and only those standing further away, managed to stay alive but suffered lower extremity injuries due to the ballistic angle of flight (24).

Peleg et al. previously described that apart from chest, spine, and abdominal injuries, which are more frequent in GSW victims, all other body regions are injured more frequently in explosions. The involvement of multiple body regions in a single patient is significantly more common in explosion victims, 62% compared with 47% in GSW patients (16). Previous reports found that the chest was the single most frequently injured body region for both SB and GSW, while the abdomen was the most frequently injured body region secondary to GSW (16).

According to our data, almost two-thirds of the TVT patients were injured in the head and lower extremities. Similar findings were previously described by Peleg (16). This is potentially explained by the fact that the majority of explosions occurred in confined spaces such as buses and shops.

Our data indicate that the lower extremities were the single most frequently injured body region. Although the exact cause of lower extremities injury is unknown, it makes sense that most of those injuries were secondary to explosions. We should remember that a sniper takes aim at the victim’s torso, i.e., the center of the body mass. The same implies to the SB, wearing a suicide vest on his chest, which creates an explosion wave beginning at the torso height and as such mainly affects the upper part of the body. On the other hand, explosions of an IED, left on the floor of a coffined place (restaurant, café) while the victims are sitting, will mainly injure the lower part of the body.

The fact that many of the VT injuries were extremity injuries is of crucial importance to the management of the patients, as it simplifies the diagnostic approach.

As was shown by Frykberg and others, the only diagnostic procedure that should be performed is a proper physical examination, which dictates either immediate intervention, when there are “hard signs” of vascular injury, or conservative approach in the setting of “soft signs” (25). The importance of this simple approach cannot be over emphasized, especially in the multi-casualties incident (MCI), during terrorist attack, when the resources are limited and should not be wasted unnecessarily.

## Conclusion

To conclude, the injuries of victims of terrorist attacks tend to be more complex than the non-TT injuries. From the data we presented, it is evident that in case of terrorism injuries, which generate a high rate of vascular injuries (10%) a vascular surgeon should be an integral part of the core trauma team, which is currently composed of general surgeons and critical care personnel, with an orthopedic surgeon and neurosurgeon on standby. A vascular surgeon as a part of the core trauma team will enable an efficient clinical approach to the injured patients.

## Author Note

Congresses: the data were presented at the 38th Veith Symposium in New York, 2011.

## Conflict of Interest Statement

The authors declare that the research was conducted in the absence of any commercial or financial relationships that could be construed as a potential conflict of interest.
